# Behcet disease combined with Sjogren syndrome: A unique case report and literature review

**DOI:** 10.1097/MD.0000000000010138

**Published:** 2018-03-23

**Authors:** Fang-He Ju, Ting-Zhen Xu, Hui-Hua Hong, He Mao, Meng Wang, Zhen Wang

**Affiliations:** a Department of Respiratory Medicine, The First Affiliated Hospital of Zhejiang Chinese Medical University; b Department of Respiratory Medicine, Zhejiang Provincial Hospital of Traditional Chinese Medicine; c Department of Dermatovenereology Medicine, The First Affiliated Hospital of Zhejiang Chinese Medical University; d Department of Dermatovenereology Medicine, Zhejiang Provincial Hospital of Traditional Chinese Medicine, Hangzhou, Zhejiang, People's Republic of China.

**Keywords:** analysis, Behcet disease, case study, review, Sjogren syndrome

## Abstract

**Rationale:**

Behcet disease(BD) and Sjogren syndrome(SS) are separate conditions that rarely concomitantly affect an individual. In theory,mild symptoms of patients with BD or SS are easy to igore and,thus,remain undiagnosed. There,it is reasonable to believe there may be some clinical cases of combined diseases that go undiscovered and which needs to be taken seriously. In addition,it has been suggested that herpes simplex virus(HSV) types 1 and 2 are associated with BD,but have not been shown to be correlated to the direct pathogenesis of BD. The role of HSV in BD needs more research and attention.

**Patient concerns:**

Here,we report a young woman who had both BD and SS. The first symptom of the disease was fever. However,the HSV type 1 IgG and HSV type 2 IgM antibody results were positive in our case and,which rendered this case unique.

**Diagnoses:**

BD and SS concomitantly affect the individual,and BD was the acute type.

**Interventions:**

IV methylprednisolone was used for 9 days and then oral glucocorticoids was used to instead,and the treatment works very well.

Outcomes: BD and SS can concomitantly affect an individual,and we believe that HSV-2 may be directly related to the pathogenesis of BD. The nature of BD as an auto-inflammatory disorder, autoimmune disorder, or both, is controversial. If we can find more patients who combined affected these two disease, it might helpful for us to understand the nature of BD.

**Lessons:**

For patients with clinical diagnosis of BD or SS,we need to be alert that it may combinded the other disease. Long term follow up and detailed inspection are important means to avoid undiscovered.

## 1 Introduction

Behcet disease (BD) is a chronic form of relapsing multisystem vasculitis characterized by recurrent oral and genital ulcers, with ocular and skin involvement.^[1]^ BD can be divided into acute and chronic types, the former being rare.^[2]^ The etiology of BD is unknown.^[3]^ Sjogren syndrome (SS) is a chronic, systemic, autoimmune disease characterized by focal lymphocyte infiltration in the exocrine glands, particularly the salivary and lacrimal glands.^[4]^ BD and SS rarely concomitantly affect an individual. Indeed, only a few cases have been reported.^[5–9]^ In these case reports, none of the patients had acute fever as the initial symptom of BD, and none of the reported cases involved Chinese patients.^[5–9]^ In one of these cases, the pathological diagnosis of SS was suspected.^[6]^ In another,^[8]^ according to the information provided, SS could not be confirmed via the European Study Group on Classification Criteria for SS.^[10]^ In another case report, no detailed clinical data were provided.^[9]^ Therefore, there are limited confirmed case reports that include detailed clinical data concerning combined BD and SS.

Here, we report a Chinese woman who was simultaneously diagnosed with BD (acute type) and SS, in which fever was the initial symptom of BD.

## 2 Case report

A 39-year-old woman presented with a fever. After the fever had persisted for 3 days, she began using cefdinir, but her temperature continued to increase, reaching 40.3°C. She was hospitalized after the fever had persisted for 5 days. She had a history of penicillin allergy and had previously undergone abortions at 28 and 38 years of age. From 31 years of age, she had taken contraceptive drugs for 1.5 years. At the age of 34 years, she had undergone a cesarean section. Since the age of ∼36 years, she had experienced dry eyes, but had felt that the symptoms were tolerable. She had occasionally used eye drops to relieve her dry eyes. She had no obvious oral or vaginal dryness, and no family history of genetic or autoimmune disease. During a comprehensive physical examination, an enlarged lymph node (3.2 × 1.1 cm) was detected in the right inguinal region.

Standard laboratory blood analysis results revealed a white blood cell (WBC) count of 8.2 × 10^9^/L (3.5–9.5 × 10^9^/L), red blood cell count of 3.89 × 10^12^/L (3.80–5.10 × 10^12^ /L), hemoglobin level of 123 g/L (115–150 g/L), platelet count of 136 × 10^9^/L (125–350 × 10^9^ /L), and C-reactive protein level of 69 mg/L (0–8 mg/L). Test results were negative for human immunodeficiency virus, hepatitis B and C virus, herpes simplex virus (HSV) type 1 IgM, cytomegalovirus IgM, Coxsackie virus IgM, adenovirus IgM, Epstein–Barr virus IgM, respiratory syncytial virus IgG and IgM, and anticyclic citrullinated peptide antibody, as was the result for the Widal test. Test results were positive for HSV type 1 IgG, HSV type 2 IgM and IgG, cytomegalovirus IgG, Coxsackie virus IgG, adenovirus IgG, and Epstein–Barr virus IgG. A rheumatoid factor test yielded a result of 83.75 IU/mL (normal range 0–40 IU/mL), and the erythrocyte sedimentation rate was 93 mm/hour (normal range 0–20 mm/hour). Anti-streptolysin O, complement C3, and complement C4 results were all normal. Computed tomography scanning of the lungs revealed normal findings, and no abnormalities of the liver, gallbladder, pancreas, spleen, or kidneys were detected via ultrasound. The disease history is summarized in Table 1.

**Table 1:**
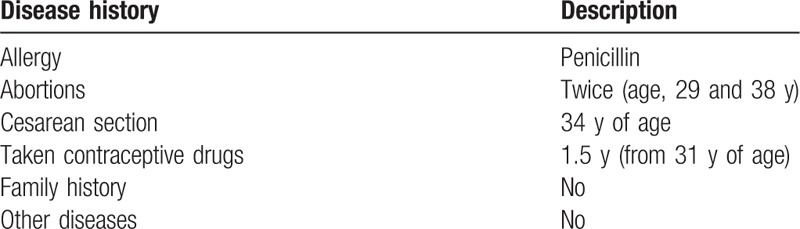
Patient disease history.

From the first day after hospitalization, the patient received intravenous (IV) levofloxacin 0.5 g once daily. On the second day, red papules and partial papule surface pustules appeared over her entire body, and she reported slightly blurred vision. The fever persisted, and the red papules spread. On the third day, we added 2 g IV cefmetazole sodium to her treatment regimen, but her temperature continued to increase, and the rash continued to worsen. On the fourth day after hospitalization, multiple painful ulcers surrounding the lingual frenum and labia minora rapidly appeared. Papulopustular lesions spread over her body and were particularly prominent on her abdomen and legs. After changing her treatment to teicoplanin on the fifth day after hospitalization, her condition failed to improve. She denied any history of oral or labial ulcers. A labial gland biopsy indicated gland atrophy with fibrosis and interstitial fat metaplasia accompanied by infiltration of chronic inflammatory cells. Histological features included a positive biopsy of a minor salivary gland, which is defined as a focus score >1 and refers to a cluster of more than 50 lymphocytes per lobule when at least 4 lobules are assessed. Test results were positive for antinuclear antibodies, anti-Sjogren syndrome antigen A (SS-A) antibodies, anti-Sjogren syndrome antigen B (SS-B) antibodies, and Ro-52. Results for antineutrophil cytoplasmic antibodies were negative. On the sixth day after hospitalization, we performed an eye examination and the tear film breakup time was less than 1 second in both eyes. A Schirmer paper-strip tear test was positive. Bilateral retinal scattered seepage lesions were detected. Physical examination and biochemical test results are described in Table 2.

**Table 2:**
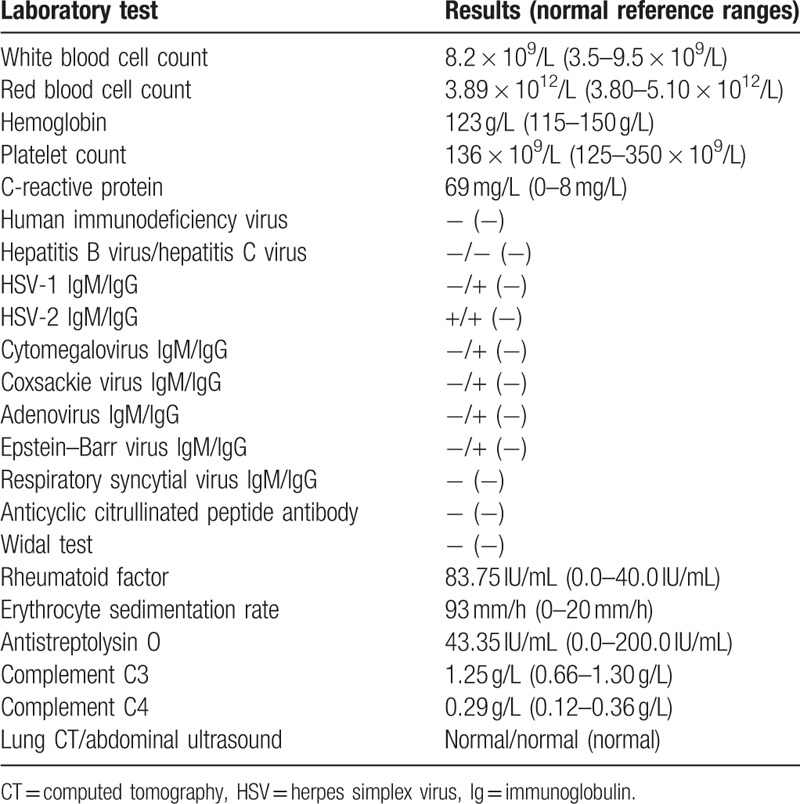
Clinical and laboratory findings.

**Figure 1:**
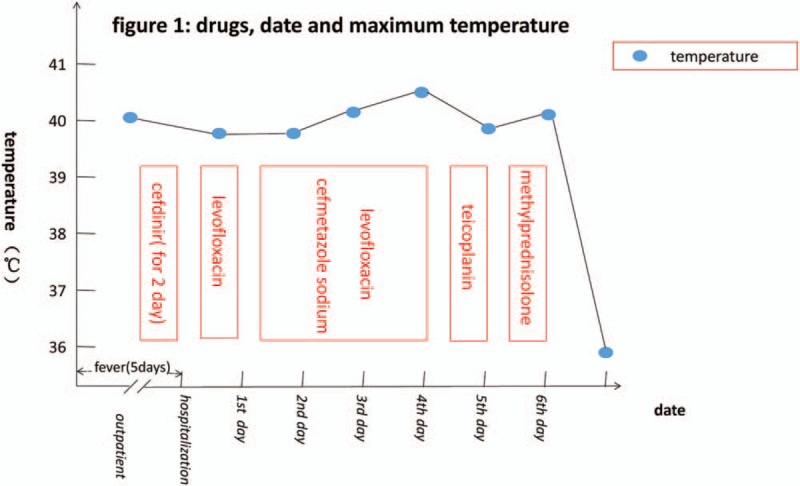
Drugs, date, and maximum temperature.

Given these results, the patient was diagnosed with BD per the criteria of the International Study Group for Behcet's Disease.^[11]^ She was also diagnosed with SS, based on the criteria of the European Study Group on Classification Criteria for Sjogren's Syndrome.^[10]^ On the sixth day after hospitalization, we changed her treatment to IV cefuroxime 3 g twice a day (for 4 days) and IV methylprednisolone 40 mg/day. Three days later, the methylprednisolone dose was reduced to 30 mg/day for another 6 days and oral prednisone was used instead of IV methylprednisolone. The patient's condition improved markedly. She was discharged from the hospital and, after treatment with oral glucocorticoids for 1 week, the papules completely faded, and the oral and labial ulcers improved markedly, as did the blurred vision. At a 2-month follow-up, the bilateral retinal scattered lesions and seepage were nearly completely resolved, and the patient reported no discomfort. The treatment process is shown in Fig. 1.

The institutional ethics committee of the Zhejiang Provincial Hospital of Traditional Chinese Medicine approved the present study. Patient consent was given.

## 3 Discussion

BD combined with SS is considered rare. To the best of our knowledge, this is the first Chinese patient in whom both conditions have been reported. Additionally, among the cases of combined BD and SS reported to date, the current case is the first in which the primary initial symptom of BD was fever.

Moreover, there are other differences in our case when compared with other reported cases.^[5–9]^ There was an earlier report of an elderly Japanese woman with BD combined with SS^[5]^; however, antinuclear antibodies, anti-SS-A antibodies, and anti-SS-B antibodies were not detected in the patient's serum. In addition, the patient in the current case was younger and had no hypochromic microcytic anemia, unlike the aforementioned Japanese patient. We also recorded detailed information about the patient's acute onset process, such as the appearance of papules, and her responses to treatment, which were not mentioned in the previous report.^[5]^ In another previous report of a female Japanese patient with suspected SS, BD, and rheumatoid arthritis,^[6]^ the patient's antinuclear antibody test results were positive, while tests for anti-SS-A and anti-SS-B antibodies were negative. Moreover, her pathological diagnosis was suspected, rather than conclusive. A Caucasian man with BD combined with SS has been reported.^[7]^ This patient tested positive for antinuclear, anti-SS-A, and anti-SS-B antibodies. Another case of combined BD and SS was reported in the Lancet more than 40 years ago,^[8]^ but the information provided in that report is limited. In fact, according the data in that report,^[8]^ SS could not be confirmed in that patient via the European Study Group on Classification Criteria for SS.^[10]^ A retrospective review of 340 cases with joint manifestations identified 601 cases of BD, of which 2 also had SS^[9]^; however, there was a lack of detailed and specific data reported about these 2 cases.

The current report of a case of combined BD and SS includes detailed information and definitive diagnoses. The acute type of BD is relatively rare, and a case combined with SS is even rarer.

Considering that small oral ulcers are easy to ignore, BD may go undiagnosed in some patients. Mild symptoms of SS may also, in theory, be unnoticed by patients and doctors, leading to a missed diagnosis. Therefore, in this case analysis, we primarily emphasize that it is reasonable to believe that there may be more undiscovered clinical cases of combined BD and SS.

Currently, HSV is the only virus for which a possible association with BD has been speculated.^[1,12]^ Notably, serum anti-HSV type 1 antibodies were reportedly found in a higher proportion of patients with BD than in controls.^[13]^ However, there is also research that HSV-1/2 is associated with BD, but not with the direct pathogenesis of BD, since HSV-1 and HSV-2 genomic material is also detected in other related inflammatory disease samples of lesion tissue.^[14]^ HSV is most likely implicated as BD through an influence on T-cell immunoregulation.^[15]^ However, in this case, we speculated that HSV-2 is directly correlated to the pathogenesis of BD because HSV type 2 IgM antibody and HSV type 1 IgG antibody results were positive, and BD, in this case, was the acute type. Therefore, we believe that HSV-2 is directly correlated to the pathogenesis of BD.

Although the patient's C-reactive protein levels were higher than normal, the use of antiinfective drugs for many days was ineffective, and her condition worsened. In contrast, after the initiation of treatment with glucocorticoids, her symptoms improved rapidly. There are limited data regarding the therapeutic effects of antiviral treatment in BD, and the results are controversial.^[1,12]^ For this reason, we did not administer antiviral treatment. Rather, we administered glucocorticoids, which produced a remarkable effect. For the patient in this case, antiviral therapy may not be critical in acute patients.

Furthermore, as previously mentioned, when BD is combined with SS, one of them may be undiagnosed if the symptoms are mild. We also speculate that the 2 diseases may be associated, as reported previously.^[7]^

Ultimately, the nature of BD as an autoinflammatory disorder, autoimmune disorder, or both, is controversial.^[16]^ Nevertheless, the study of BD in combinations with SS is useful in elucidating the nature of both conditions and understanding if the 2 syndromes are truly related.

## 4 Author contributions

**Conceptualization:** F.H. Ju, T.Z. Xu, H.H. Hong, H. Mao, M. Wang, Z. Wang.

**Data curation:** F.H. Ju, T.Z. Xu, H.H. Hong, H. Mao, M. Wang, Z. Wang.

**Formal analysis:** F.H. Ju, T.Z. Xu, H.H. Hong, H. Mao, M. Wang, Z. Wang.

**Funding acquisition:** H.H. Hong, Z. Wang.

**Investigation:** H. Mao, M. Wang, Z. Wang.

**Methodology:** H. Mao, M. Wang, Z. Wang.

**Project administration:** Z. Wang.

**Resources:** F.H. Ju, T.Z. Xu, H.H. Hong, H. Mao, M. Wang, Z. Wang.

**Supervision:** T.Z. Xu, H.H. Hong, H. Mao, Z. Wang.

**Validation:** H. Mao, M. Wang, Z. Wang.

**Visualization:** T.Z. Xu, H.H. Hong, H. Mao, M. Wang, Z. Wang.

**Writing – original draft:** F.H. Ju.

**Writing – review & editing:** F.H. Ju, Z. Wang.

## Acknowledgment

The authors wish to thank Editage (www.editage.cn/) for English language editing.
